# Disruption of methionine synthesis repressor makes *Escherichia coli* mutualistic to host stinkbug

**DOI:** 10.1128/mbio.03883-25

**Published:** 2026-01-30

**Authors:** Yayun Wang, Ryuichi Koga, Minoru Moriyama, Takema Fukatsu

**Affiliations:** 1 Molecular Biosystems Research Institute, National Institute of Advanced Industrial Science and Technology (AIST) 73459, Tsukuba, Japan; 2 Department of Biological Sciences, The University of Tokyo, Tokyo, Japan; 3 Graduate School of Life and Environmental Sciences, University of Tsukuba 13121, Tsukuba, Japan; Georgia Institute of Technology, Atlanta, Georgia, USA

**Keywords:** *Plautia stali*, *Escherichia coli*, methionine, essential amino acid, experimental evolution, symbiosis

## Abstract

Degenerative genome evolution is widely found among obligatory bacterial mutualists, as observed in plant-sucking hemipteran insects whose symbiont genomes are highly reduced and specialized for provisioning of essential amino acids. Originally, such symbionts must have been derived from environmental free-living bacteria. It is elusive, however, what evolutionary changes are involved in the early stages of such elaborate mutualistic associations. Here, we addressed this evolutionary question using the experimental symbiotic system consisting of the stinkbug *Plautia stali* and the model bacterium *Escherichia coli*. In *E. coli*, *metJ* encodes a repressor of the methionine synthesis pathway, and its disruption upregulates production of the essential amino acid methionine. We found that, when *metJ*-disrupted *E. coli* was inoculated to *P. stali*, the insects exhibited significantly elevated hemolymphal methionine levels and improved adult emergence rates, demonstrating that the single-gene mutation makes *E. coli* mutualistic to *P. stali*. In comparison with mutualistic *E. coli* single-gene mutants that upregulate another essential amino acid tryptophan, the phenotypic effects on *P. stali* were somewhat different: the adult emergence rate was improved by both the methionine-overproducing and tryptophan-overproducing *E. coli* mutants, whereas the adult body color was improved by the tryptophan-overproducing *E. coli* mutant only. When we generated a double mutant *E. coli* Δ*metJ*Δ*tnaA* and inoculated it to *P. stali*, the adult emergence rate was not improved but rather attenuated, uncovering non-additive fitness consequences of these single-gene mutations. These results provide insights into what genetic changes may have facilitated the early evolution of the insect-microbe mutualism.

**IMPORTANCE:**

What is the evolutionary origin of elaborate bacterial mutualists entailing drastic genome reduction, specialized metabolism, and uncultivability? This question is important but challenging to address, because the evolution of such symbiotic associations occurred in the past and cannot be observed directly. However, the recent development of an experimental symbiotic system consisting of the stinkbug *Plautia stali* as host and the model bacterium *Escherichia coli* as symbiont has opened an avenue to empirically investigate the evolution of host-microbe mutualism. We demonstrated that, strikingly, single-gene mutations of *E. coli* that upregulate the production of methionine and tryptophan make the non-symbiotic bacterium mutualistic to *P. stali*, plausibly via provisioning of the essential amino acids that complement the nutritional requirements of the plant-sucking insect host. Our finding provides insight into what genetic changes of the symbiont side can be involved in the early evolution of the host-microbe mutualism.

## INTRODUCTION

Nutritional symbiosis has been demonstrated to play pivotal roles in the survival and evolution of many insect species, wherein microbial symbionts supplement essential nutrients, such as amino acids or vitamins, that are scarce in their natural diet (1–3). For example, most insects of the order Hemiptera are obligatorily associated with specific microbial partners, because their sole food source, plant sap, contains little proteins and therefore must be nutritionally supplemented by symbiont-produced essential amino acids (3–5). In aphids, the endocellular bacterial symbiont *Buchnera* is specialized for provisioning of essential amino acids at the genomic level, in which the symbiont genome has lost many genes during the evolutionary course, resulting in a drastically reduced genome size (less than 1/6 of allied free-living bacterial genomes) but retaining the majority of synthesis pathway genes for essential amino acids (6–9). In many *Buchnera* strains, synthesis pathway genes for some essential amino acids, such as tryptophan and leucine, are encoded and amplified on plasmids (10–13). Such an evolutionary pattern toward genome reduction streamlined for provisioning of essential amino acids is commonly found not only among bacteriocyte-associated endosymbiotic bacteria of aphids, whiteflies, cicadas, leafhoppers, and others, but also among gut-dwelling extracellular symbiotic bacteria of stinkbugs (14–17).

Many plant-sucking stinkbugs are associated with specific symbiotic bacteria of mutualistic nature in a posterior region of the midgut, where numerous sac-like structures, called crypts, host a bacterial population within the inner cavity extracellularly (16, 18–20). In some stinkbug groups (e.g., Plataspidae and Urostylididae), their gut symbiotic bacteria have drastically reduced genomes specialized for provisioning of essential amino acids, which are parallel to *Buchnera* in aphids (21–23), whereas in other stinkbug groups (e.g., Pentatomidae and Scutelleridae), their gut symbiotic bacteria exhibit various levels of genome reduction, cultivability, and metabolic capacity (24–28). Comparative genomic and functional analyses of these stinkbug symbionts have provided some clues to understanding how reductive genome evolution toward the specific symbiotic function proceeds under host’s sap-sucking physiology and ecology (16, 25–31).

Here, a totally unaddressed question remains—What evolutionary changes are involved in early evolutionary stages of such host-symbiont mutualistic associations? Recently, an experimental evolutionary system consisting of the stinkbug *Plautia stali* as host and the model bacterium *Escherichia coli* as symbiont was developed, which enabled empirical studies on mutational events and molecular mechanisms at an early stage of the evolution of mutualistic symbiosis (32–35). Previous studies using the system uncovered that (i) even a single-gene disruption (e.g., *cyaA* encoding adenylate cyclase and *crp* encoding cyclic AMP receptor protein) can make *E. coli* mutualistic to *P. stali* (32), (ii) downregulation or disruption of a single-gene *tnaA*, which encodes a tryptophan-degrading enzyme (= tryptophanase), can make *E. coli* mutualistic to *P. stali* with elevated intra-host tryptophan levels (35), and therefore (iii) the symbiont-mediated upregulation of an essential amino acid, tryptophan, may have played a pivotal role at an early evolutionary stage of the insect-bacterium mutualism.

Here, we report the discovery that another essential amino acid, methionine, can also contribute to the establishment of *P. stali-E. coli* mutualism. We found that *P. stali* infected with the original bacterial symbiont exhibited methionine levels over 10 times higher than those infected with symbiotic and non-symbiotic *E. coli* strains and demonstrated that upregulated methionine production by disruption of a single gene, *metJ*, which encodes a repressor of the methionine synthesis pathway, makes *E. coli* improve adult emergence of the infected host *P. stali*.

## RESULTS AND DISCUSSION

### Original symbiont infection entails drastically higher methionine levels than symbiotic *E. coli* infection in *P. stali*

**Fig 1:**
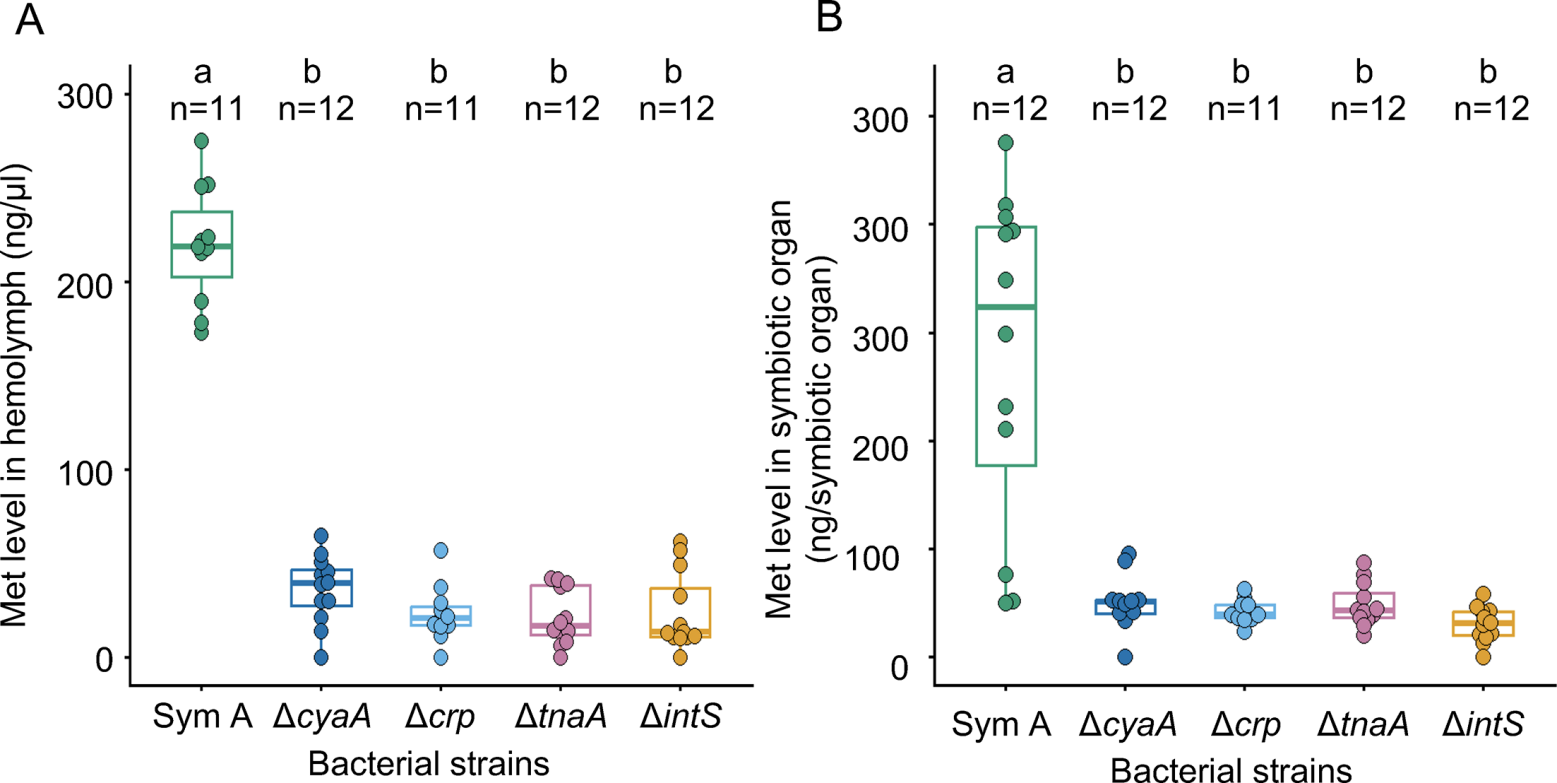
Methionine levels in hemolymph (**A**) and symbiotic organ (**B**) of *Plautia stali* infected with a natural symbiont *Pantoea* sp. A (SymA), mutualistic mutant *Escherichia coli* strains (Δ*cyaA*, Δ*crp,* and Δ*tnaA*), and a control non-symbiotic *E. coli* strain (Δ*intS*). Different alphabetical letters (a, b) indicate statistically significant differences (pairwise Wilcoxon rank-sum test with Hommel’s correction: *P* < 0.05).

We conducted a series of metabolomic comparisons between the insects infected with the original symbiont SymA (25), the mutualistic *E. coli* strains Δ*cyaA*, Δ*crp*, and Δ*tnaA* (32, 35), and a control non-symbiotic *E. coli* strain Δ*intS*. In addition to the significant difference in tryptophan levels as mentioned above, we also found a notable difference in another essential amino acid, methionine. Methionine levels in hemolymph and symbiotic organ of the SymA-infected insects were about 10 times higher than those of the insects infected with the mutualistic as well as the non-symbiotic *E. coli* strains (Fig. 1; Fig. S1). This finding prompted us to investigate whether methionine upregulation in *E. coli* affects its symbiotic capability to *P. stali*.

### Infection with *metJ*-disrupted *E. coli* results in global upregulation of methionine biosynthesis genes and elevated methionine level in *P. stali*

**Fig 2:**
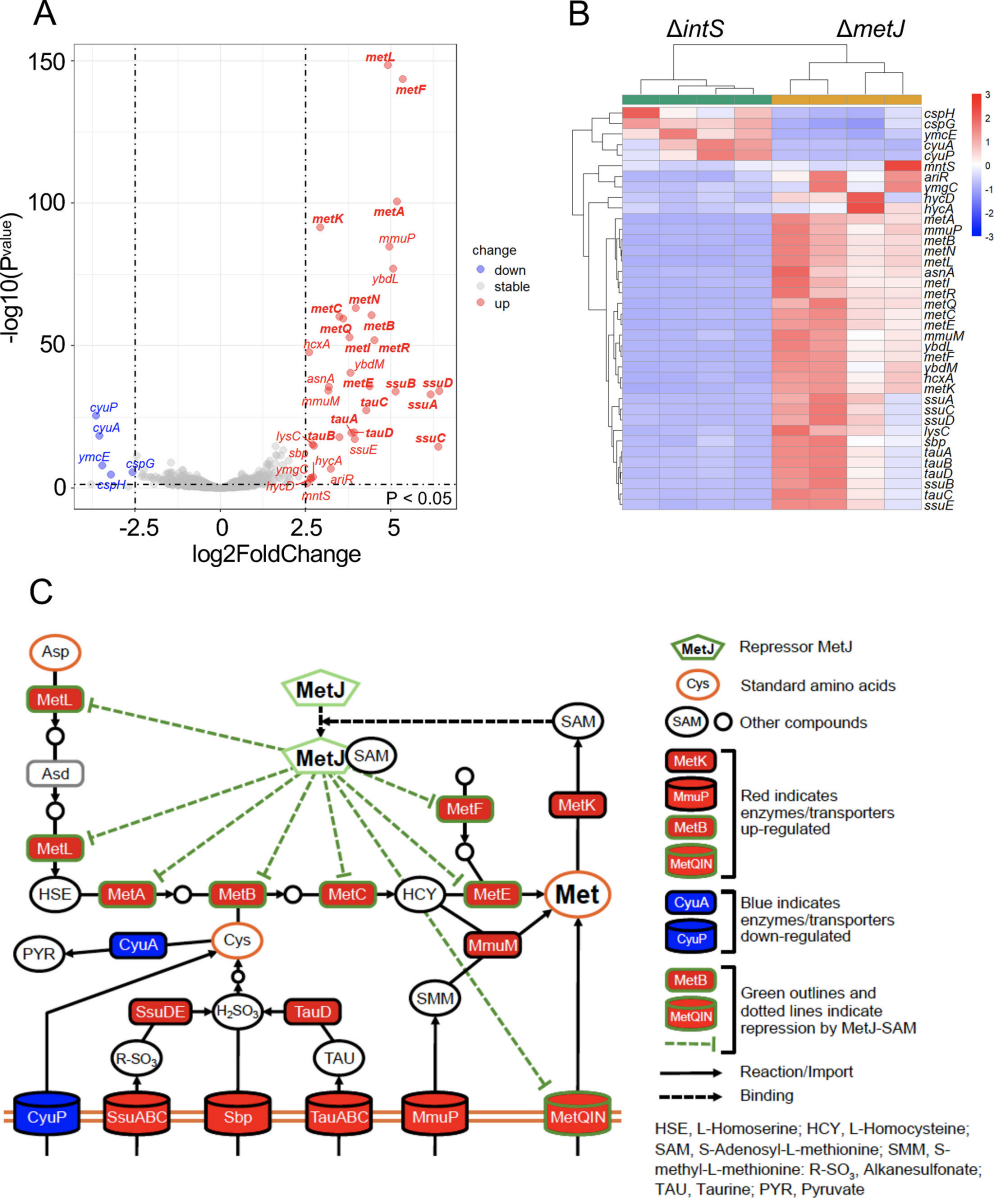
Comparative transcriptomic analyses between the *Escherichia coli* strains Δ*metJ* and Δ*intS* infecting the symbiotic organ of *Plautia stali*. (**A**) Volcano plot of differentially expressed genes (DEGs) using *E. coli*-derived reads. Red dots (upregulated), significantly upregulated 33 genes (log_2_ FC ≥ 2.50, *P* < 0.05); blue dots (downregulated), significantly downregulated 5 genes (log_2_ FC ≤ −2.50, *P* < 0.05); gray dots (stable), 3,832 genes below the significance threshold. (**B**) Cluster analysis of the 38 DEGs (|log_2_ FC| ≥ 2.50, *P* < 0.05), which is based on the transcriptomic data from four Δ*intS*-infected insects and four Δ*metJ*-infected insects. The color chart depicts the levels of upregulation (red) or downregulation (blue) of the genes. (**C**) Metabolic pathways related to methionine biosynthesis in *E. coli*. Display items are explained on the right side. The upregulated genes are highlighted in red, whereas the downregulated genes are shown in blue. Green outlines and dotted lines indicate repression by MetJ-SAM.

**Fig 3:**
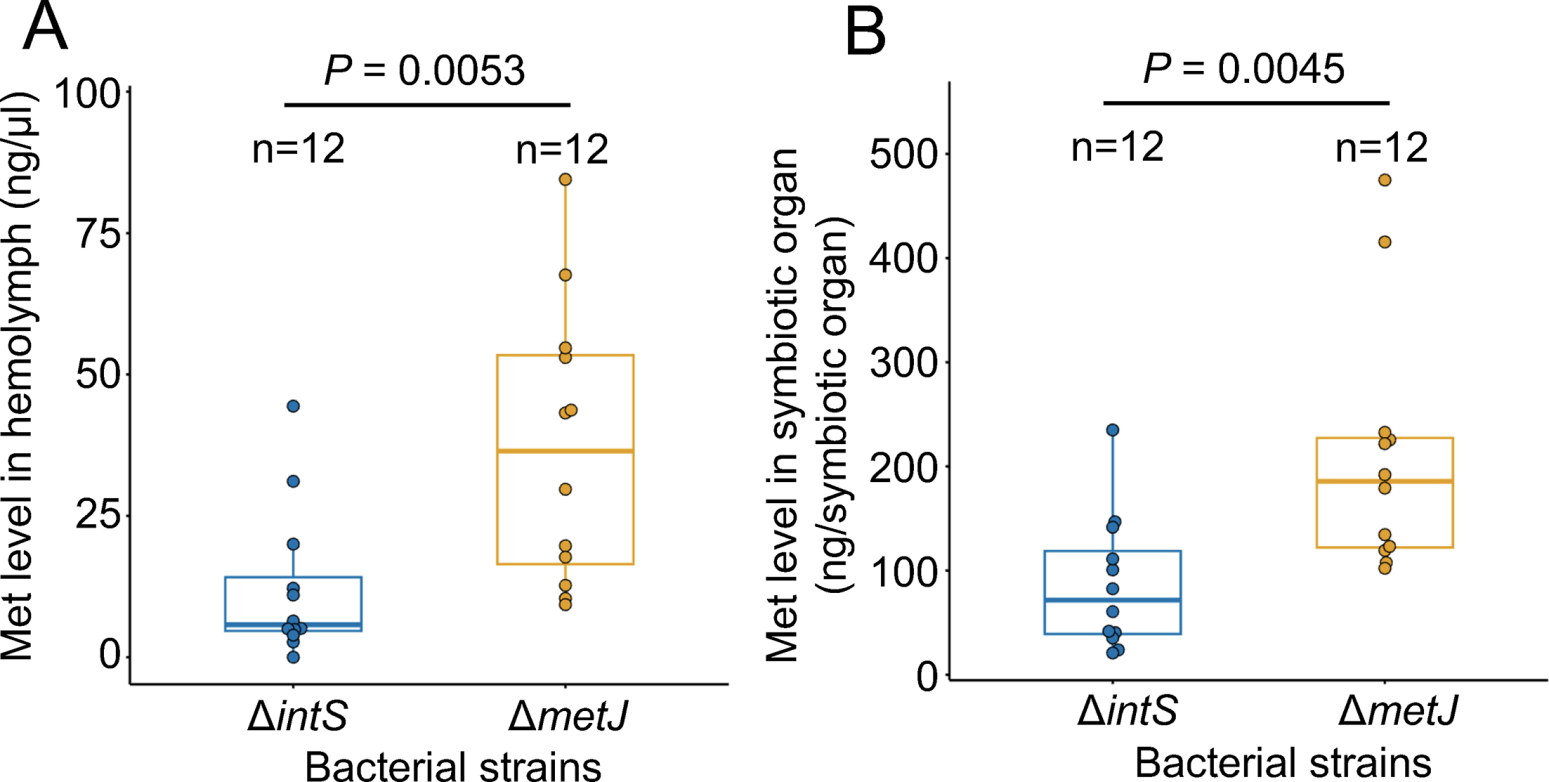
Free methionine levels in adult insects of *P. stali* infected with the *E. coli* strains Δ*intS* and Δ*metJ*. (**A**) Methionine levels in hemolymph. (**B**) Methionine levels in symbiotic organ. Statistical analysis was conducted by Student’s *t*-test.

In *E. coli*, methionine biosynthesis is mainly mediated by the trans-sulfurylation pathway, wherein homoserine is first converted to O-succinyl-l-homoserine by an enzyme MetA, then to cystathionine by an enzyme MetB, next to homocysteine by an enzyme MetC, and finally to methionine by a methylation enzyme MetE (36). The methionine synthesis pathway is regulated at the transcriptional level by a repressor protein MetJ. When methionine levels are high, its derivative S-adenosylmethionine (SAM) accumulates and acts as a co-suppressor, which enhances affinity of MetJ to specific DNA sequences, called Met boxes, in the promoter regions of methionine biosynthetic genes. The MetJ-SAM complex binds these sites, thereby repressing transcription and regulating methionine synthesis (37). Hence, disruptive mutations in *metJ* lead to de-repression of the methionine biosynthetic pathway, resulting in constitutive high expression of methionine biosynthesis enzymes and elevated methionine production (38). We inoculated Δ*metJ* and Δ*intS E. coli* strains to newborn nymphs of *P. stali*, the nymphs were reared to adulthood, and the adult insects were subjected to dissection of their symbiotic organ for transcriptomic analysis. Comparative transcriptomics of *E. coli*-derived sequence reads from the symbiotic organs revealed that, of 3,870 *E. coli* genes identified, 33 genes were significantly upregulated and 5 genes were significantly downregulated for Δ*metJ* in comparison with Δ*intS* under the intra-host condition (Fig. 2A and B; Table S1). The majority of the upregulated genes were related to methionine biosynthesis/utilization (*metABCEFKLR*) and methionine/sulfur compound transport (*metNQI*, *ssuABCDE*, *tauABCD*, and *sbp*), whereas the downregulated genes were related to cysteine metabolism (*cyuPA*) (Fig. 2C; Table S1). Amino acid analysis of hemolymph and dissected symbiotic organs confirmed significantly higher methionine levels in the Δ*metJ*-infected insects in comparison with the Δ*intS*-infected control insects (Fig. 3; Fig. S2).

### Infection with *metJ*-disrupted *E. coli* improves adult emergence of *P. stali*

**Fig 4:**
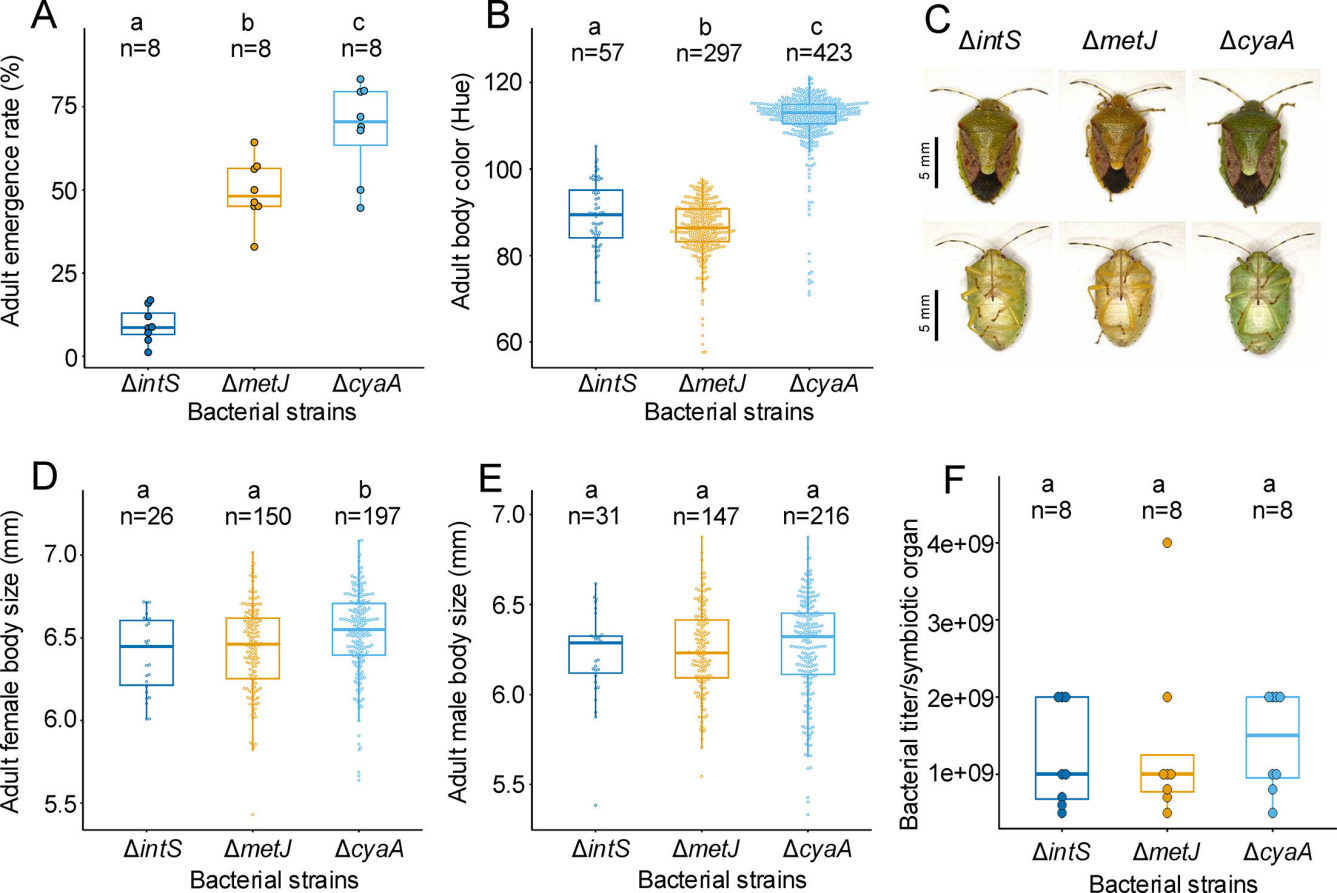
Phenotypes of *P. stali* infected with the methionine-overproducing *E. coli* strain Δ*metJ* in comparison with those infected with the carbon catabolite repression-disrupted, tryptophan-overproducing *E. coli* strain Δ*cyaA* and the non-symbiotic *E. coli* strain Δ*intS*. (**A**) Adult emergence rate. (**B**) Adult body color. (**C**) External appearance of adult insects. (**D**) Adult female body size. (**E**) Adult male body size. (**F**) Bacterial titer in the symbiotic organ in terms of *KmR* gene copies per organ. Different alphabetical letters (a, b, c) indicate statistically significant differences (pairwise Wilcoxon rank-sum test with Hommel’s correction: *P* < 0.05). Adult emergence rate was calculated using 84 eggs consisting of six egg masses.

The adult emergence rates of the Δ*metJ*-infected insects were significantly higher than those of the Δ*intS*-infected insects (Fig. 4A). This result indicates that the single-gene disruption of the methionine biosynthesis repressor *metJ* can alter *E. coli* to be more beneficial to *P. stali,* which reinforces the notion that the evolution of mutualistic symbiosis can be established by single-gene mutations (32, 33, 35). On the other hand, the Δ*metJ*-infected insects exhibited no improvement of adult body color and body size in comparison with the Δ*intS*-infected insects (Fig. 4B through E), unveiling that the beneficial effect of the *E. coli* mutation may be restricted to specific host phenotypes.

### Different phenotypes of *P. stali* infected with *metJ*-disrupted *E. coli* and *cyaA*-disrupted *E. coli*

Notably, the phenotypic consequences of *P. stali* infected with Δ*metJ E. coli*, which upregulates intra-host methionine levels, were different from those of *P. stali* infected with Δ*cyaA E. coli*, which upregulates intra-host tryptophan levels. The adult emergence rate was improved both in the Δ*metJ*-infected insects and the Δ*cyaA*-infected insects (Fig. 4A), whereas the adult body color was improved to greenish in the Δ*cyaA*-infected insects, but no such improvement was observed in the Δ*metJ*-infected insects (Fig. 4B and C). Instead, the body color of Δ*metJ*-infected insects was more yellowish and less greenish even when compared with the Δ*intS*-infected insects (Fig. 4C), suggesting that, though speculative, the disruption of *E. coli metJ* may somehow interfere with pigmentation of *P. stali*. In contrast to the adult emergence rate and the body color, no significant difference was detected in the adult body size among the Δ*intS*-, Δ*metJ*-*,* and Δ*cyaA*-infected insects, except for the slight increase in the Δ*cyaA*-infected females (Fig. 4D and E). Bacterial titers in the adult insects exhibited no significant difference between Δ*intS*, Δ*metJ*, and Δ*cyaA* (Fig. 4F), suggesting that the different host phenotypes are likely due to qualitative differences of the *E. coli* strains rather than quantitative ones.

### Comparison of fitness consequences of *P. stali* infected with *metJ*-disrupted *E. coli* and *tnaA*-disrupted *E. coli*

**Fig 5:**
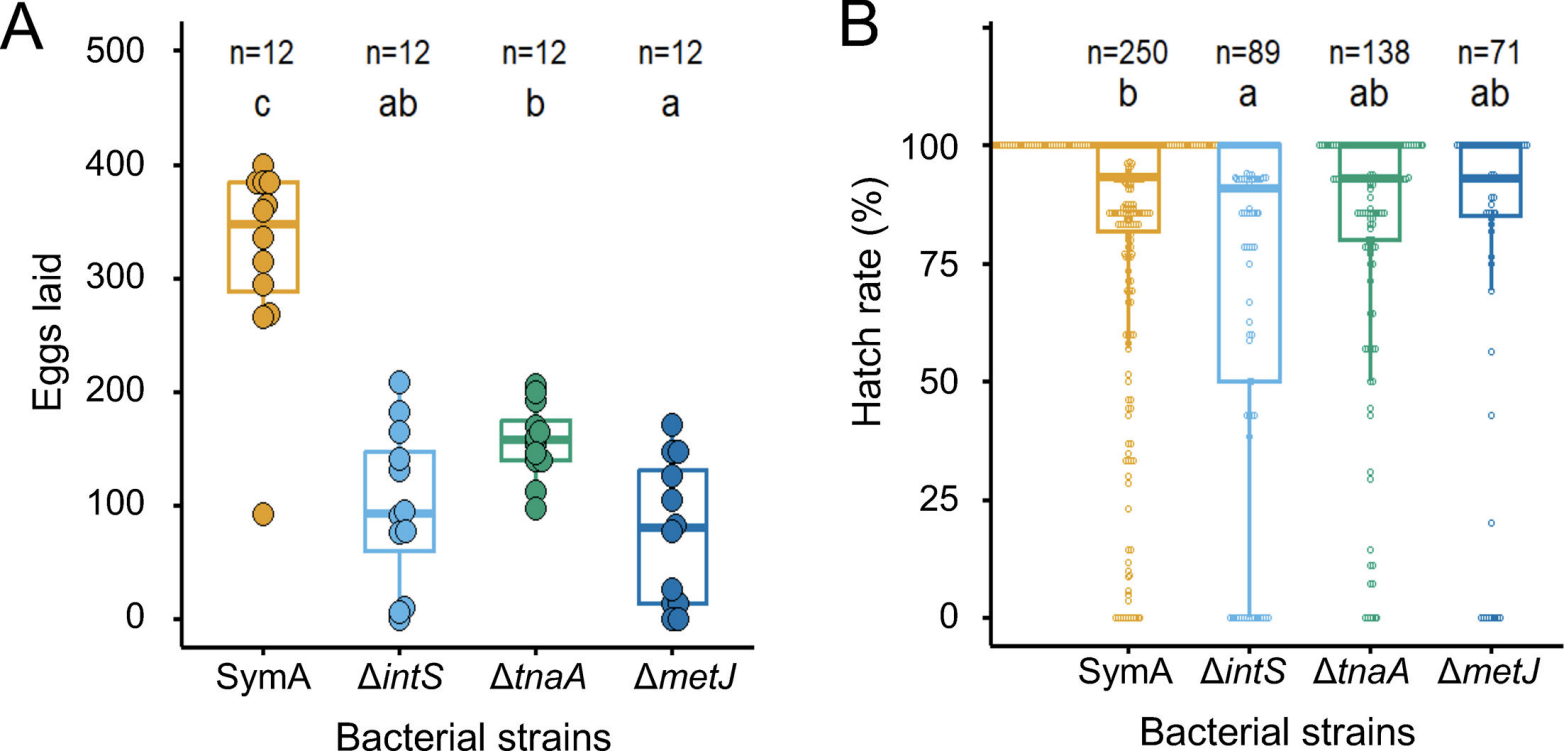
Reproductive phenotypes of *Plautia stali* infected with the methionine-overproducing *Escherichia coli* strain Δ*metJ* in comparison with those infected with the tryptophan-overproducing *E. coli* strain Δ*tnaA*, the non-symbiotic *E. coli* strain Δ*intS*, and the natural symbiont SymA. (**A**) Total number of eggs laid by each adult female during 31 days after emergence. (**B**) Hatch rate of the eggs. Both panels show box plots with individual data points and sample sizes. Different alphabetical letters (a, b, c) indicate statistically significant differences (pairwise Wilcoxon rank-sum test with Hommel’s correction: *P* < 0.05).

We recently demonstrated that the improved adult emergence rate and body color by disruption of the carbon catabolite repression component gene, *cyaA*, is ascribed to consequent suppression of a downstream gene, *tnaA* encoding tryptophanase, which results in accumulation of the essential amino acid tryptophan and reduced production of toxic indole (35). Therefore, disruption of *tnaA* rather than *cyaA* directly upregulates the intra-host tryptophan levels. We compared reproductive phenotypes of *P. stali* infected with the original symbiont SymA, the methionine-overproducing *E. coli* strain Δ*metJ*, the tryptophan-overproducing *E. coli* strain Δ*tnaA*, and the control non-symbiotic *E. coli* strain Δ*intS* (Fig. 5; Fig. S3). While the SymA-infected insects laid many eggs, over 300 per female per month on average, the *E. coli*-infected insects produced significantly smaller numbers of eggs, around 100 per female or so (Fig. 5A). Among the *E. coli* strains, Δ*tnaA* infection yielded significantly more egg production than Δ*metJ* infection (Fig. 5A). The egg hatch rates were generally high, over 90% across SymA, Δ*metJ*, Δ*tnaA*, and Δ*intS* infections, wherein Δ*intS* infection exhibited a slightly but significantly lower egg hatch rate than SymA infection (Fig. 5B). These results uncovered that (i) the *E. coli*-infected insects generally exhibit significantly lower fecundity than the insects infected with the natural symbiont SymA, (ii) on the other hand, the hatch rates of the eggs produced by the *E. coli*-infected insects were almost comparable to those of the eggs produced by the SymA-infected insects, and therefore (iii) the *E. coli*-infected insects produce normal eggs in a reduced number, which may be due to limited nutritional resources, such as essential amino acids, provisioned by the bacterial associates. The fact that the small number of eggs produced by the *E. coli*-infected insects can hatch and grow enables experimental evolutionary studies on *P. stali-E. coli* symbiosis and may predispose the laboratory symbiotic evolution toward mutualism (32, 35). A previous study reported that, in egg-producing adult insects of *P. stali*, genes for biosynthesis and transport of sulfur-containing amino acids, cysteine and methionine, are upregulated in SymA residing in the symbiotic organ, which are likely utilized for formation of cysteine-rich eggshells (39). In this study, the upregulation of methionine synthesis pathway by *metJ* disruption did not improve the egg production of the insects infected with Δ*metJ E. coli* (Fig. 5A), which may reflect the fact that upregulation of cysteine production was not observed in Δ*metJ E. coli* (see Fig. S2).

### Non-additive effects of double mutant *E. coli* Δ*metJ*Δ*tnaA* on adult emergence rate of *P. stali*

**Fig 6:**
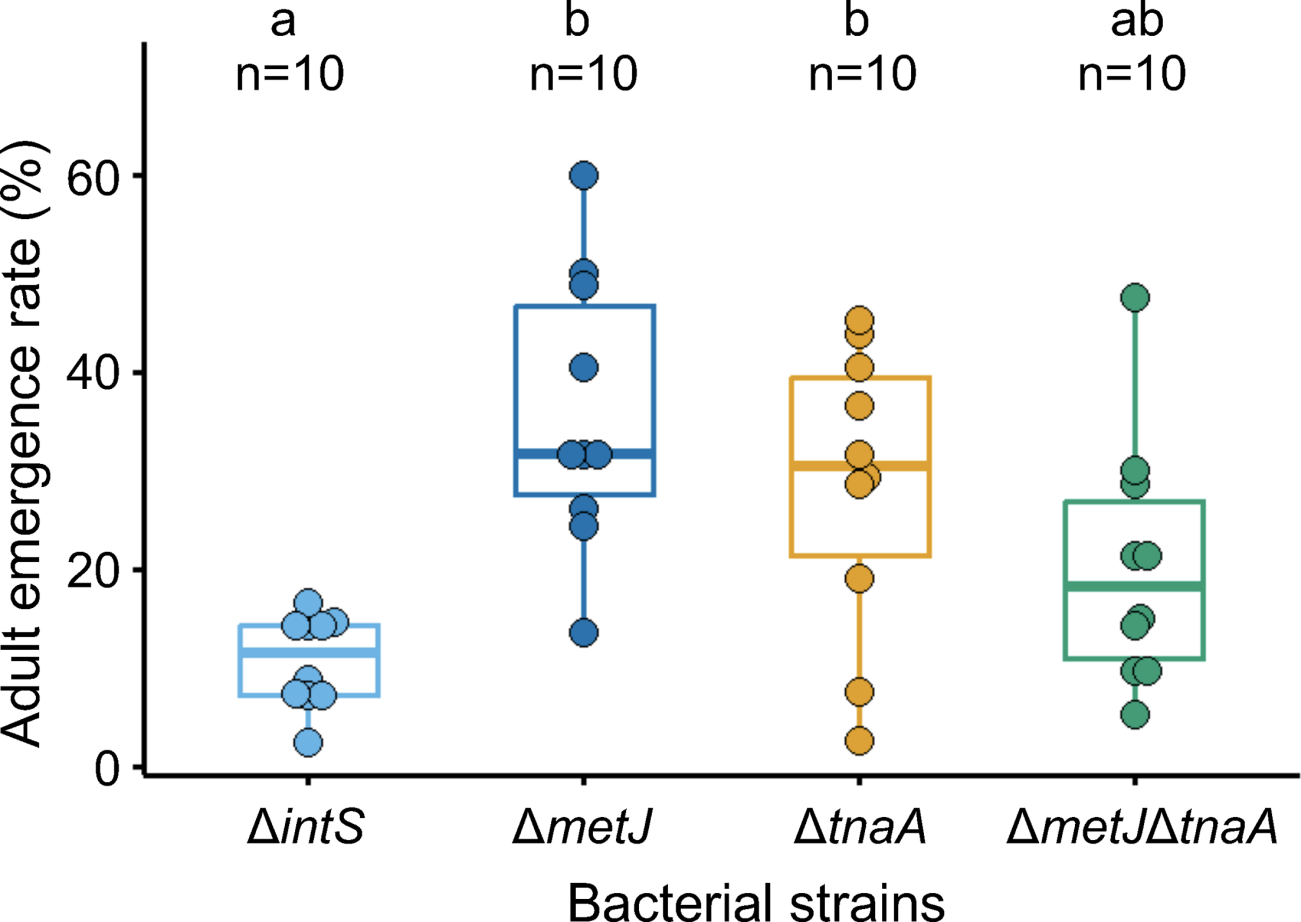
Adult emergence rates of *Plautia stali* infected with the double mutant *Escherichia coli* strain Δ*metJ*Δ*tnaA* in comparison with those infected with the Δ*metJ*, Δ*tnaA,* and Δ*intS E. coli* strains. Different alphabetical letters (a, b) indicate statistically significant differences (pairwise Wilcoxon rank-sum test with Hommel’s correction: *P* < 0.05). For each experimental group, 42 eggs consisting of 3 egg masses (except for a Δ*intS* group with 46 eggs) were used.

Finally, we generated an *E. coli* double mutant Δ*metJ*Δ*tnaA*, inoculated the mutant *E. coli* into newborn nymphs of *P. stali*, the insects were reared to adulthood, and the adult emergence rate was compared with the insects infected with the *E. coli* strains Δ*metJ*, Δ*tnaA*, and Δ*intS*. We expected that the Δ*metJ*Δ*tnaA*-infected insects would show a higher adult emergence rate than the Δ*metJ*- and Δ*tnaA*-infected insects, but actually, the adult emergence rate was lower than the Δ*metJ*- and Δ*tnaA*-infected insects, although the differences were statistically not significant (Fig. 6). These results suggested that the *metJ* disruption and the *tnaA* disruption in the same *E. coli* affect host’s adult emergence rate not in an additive manner, in which interactions between metabolic pathways for the essential amino acids methionine and tryptophan may be involved (40–43).

### Conclusion and perspective

In this study, we demonstrated that disruption of the *metJ* gene of *E. coli*, a repressor of the methionine biosynthesis pathway, results in elevated intra-host methionine levels and improved adult emergence rates of the infected host *P. stali*. Together with our previous report that elevated intra-host tryptophan levels due to disruption of the *tnaA* gene of *E. coli*, which encodes a tryptophan-degrading enzyme, also results in improved adult emergence rates of the infected host *P. stali* (35), we suggest that bacterial mutations that upregulate production/accumulation of essential amino acids may have occurred at early stages of the symbiotic evolution and facilitated the establishment of the host-symbiont mutualism. In obligatory symbiotic bacteria of plant-sucking insects, their drastically reduced genomes generally entail preferential retention of biosynthesis pathway genes for essential amino acids and, in addition, constitutive expression of metabolic genes due to loss of transcription factors and other regulatory genes (13, 44, 45). The finding that disruption of the transcription factor *metJ* makes *E. coli* mutualistic to *P. stali* may reflect such an evolution process of microbial symbiosis.

The upregulated intra-host methionine levels due to disruption of the repressor gene *metJ* and the upregulated intra-host tryptophan levels due to disruption of the tryptophanase gene *tnaA* may represent different evolutionary trajectories as to how non-symbiotic bacteria turn into symbiotic ones. It seems plausible, although speculative, that loss of genes related to the regulation/degradation of essential amino acids at early stages of symbiosis may facilitate the host-symbiont interdependence and the consequent reinforcement of the host-symbiont association.

On the other hand, when we generated the Δ*metJ*Δ*tnaA* double disruptive mutant *E. coli* and inoculated it to *P. stali*, the adult emergence rates did not improve but rather decreased in comparison with the insects infected with either Δ*metJ* or Δ*tnaA* single disruptive mutant *E. coli*. This result plausibly reflects the fact that the metabolic network of the symbiotic system is not simple but so complicated as to entail various metabolic tugs-of-war. Whether and how the other essential amino acids are upregulated and involved in the *P. stali-E. coli* mutualism will be a promising direction to be pursued in future studies.

## MATERIALS AND METHODS

### Insect samples, bacterial strains, and primers used in this study

We used an inbred laboratory strain of the brown-winged green stinkbug *P. stali* that has been maintained for approximately 100 generations. The rearing and feeding conditions were as described (32). The natural symbiont SymA of *P. stali* (25), the *E. coli* deletion mutants Δ*metJ*, Δ*tnaA*, Δ*cyaA*, Δ*crp*, and Δ*intS* (46), and the plasmids and the primers (32) used in this study are listed (Table S2).

### Insect rearing, symbiont sterilization, and bacterial inoculation

Insect rearing, symbiont sterilization, and bacterial inoculation were conducted as described (35). The insects were reared until they became adults or died, and the adult insects were subjected to recording of emergence rate, body color, and body size 6 weeks after egg collection. The adult insects were anesthetized in a refrigerator overnight, scanned from the dorsal side by a scanner (GT-X980, Epson), and the scanned images were analyzed for body size and color hue using the software Natsumushi v.1.10 (47).

### Generation of Δ*metJ*Δ*tnaA E. coli* strain

The genetic manipulation procedures were performed as described (35). To knock out the *metJ* gene of the *E. coli* strain BW25113 *ΔtnaA*, the kanamycin resistance (*KmR*) cassette was excised using FLP recombinase expressed from the plasmid pFLP3. The loss of pFLP3 was facilitated by the *sacB*-based suicide gene system, thereby creating the *E. coli* strain BW25113 *tnaA*::FRT. The successful removal of the *KmR* cassette was verified by specific PCR amplification using the primers BW25113_tnaA_F and BW25113_tnaA_R (Table S2). The primers BW25113_metJ_tn5_F and BW25113_metJ_tn5_R (Table S2) were used for PCR amplification of the *KmR* region of the control *E. coli* strain ∆*intS*. Transformation of BW25113 *tnaA*::FRT with the plasmid pRed/ET was carried out using a MicroPulser electroporator (Bio-Rad). Subsequently, the *metJ* gene in the BW25113 *tnaA*::FRT genome was replaced by the *KmR* cassette. The deletion of the *metJ* gene was confirmed by specific PCR amplification using the primers BW25113_metJ_F and BW25113_metJ_R (Table S2).

### Amino acid analysis

Hemolymph samples were collected from the neck of ice-anesthetized adult insects using 1 µL glass capillaries (Drummond). Each sample was then suspended in 100 µL of 80% (vol/vol) methanol and stored at –80°C until further analysis. Symbiotic midgut samples were dissected from adult insects, homogenized in 100 µL of 80% methanol, and stored under the same condition. Amino acid analysis was conducted by liquid chromatography and mass spectrometry as described (39).

### Quantitative PCR

Each symbiotic organ was dissected from an adult female 7 days after emergence in PBS and individually homogenized in 200 μL of sterile water. From the 200 µL homogenate, 5 µL was taken and subjected to quantitative PCR of *KmR* gene that is present in the *E. coli* strains Δ*metJ*, Δ*cyaA*, and Δ*intS*. The primers Tn5-1789F and Tn5-1879R were used for PCR amplification of *KmR* gene (Table S2). The PCR reaction was conducted using the KAPA SYBR Fast qPCR Kit (Kapa Biosystems, Belgium) and Roche LightCycler 96 System (Roche, Switzerland). A standard curve was generated with serially diluted ∆*intS* genomic DNA.

### Transcriptomic analysis

Each midgut symbiotic organ was dissected from an adult insect and individually subjected to RNA extraction using RNAiso (Takara Bio) and the RNeasy Mini Kit (Qiagen, USA), ribosomal RNA depletion using riboPOOLs (siTOOLs Biotech, Germany), cDNA library construction using TruSeq stranded mRNA Sample Preparation Kit (Illumina, USA), and DNA sequencing using HiSeq4000 (Illumina). The obtained sequences were trimmed, mapped to the *E. coli* BW25113 genome sequence (accession no. NZ_CP009273) and read-counted with CLC Genomics Workbench 22.0.2 (Qiagen). Normalization and differential expression analysis were conducted using DESeq2 package (version 1.44.0) (48). The software of the ggrepel package (version 0.9.6) (49) and the pheatmap package (version 1.0.12) (50) were used for construction of volcano plot and cluster analysis of differentially expressed genes, and to draw the maps with ggplot2 package (version 3.5.1) in R (version 4.4.0) (51).

### Statistical analysis and figure preparation

Statistical analyses were performed using R version 4.4.0 (51) and RStudio version 2025.09.2+418 (52). Images of insects were captured using a Leica S9i stereo microscope and controlled via LAS EZ Imaging software (Leica).

## Data Availability

All RNA sequencing data generated in this study have been deposited in the DNA Data Bank of Japan (DDBJ) Sequence Read Archive. The data are linked to BioProject accession number PRJDB39868 (Runs: DRR892672–DRR892679) in the DDBJ BioProject database. Source data are provided with this paper.

## SUPPLEMENTAL MATERIAL

Supplemental figures mbio.03883-25-s0001.pdf Fig. S1-S3.

Supplemental tables mbio.03883-25-s0002.xlsx Tables S1 and S2.

ASM does not own the copyrights to Supplemental Material that may be linked to, or accessed through, an article. The authors have granted ASM a non-exclusive, world-wide license to publish the Supplemental Material files. Please contact the corresponding author directly for reuse.
