# Supplementary material for: Disruption of methionine synthesis repressor makes Escherichia coli mutualistic to host stinkbug
Source: mBio. 2026 Jan 30;17(3):e03883-25. doi: 10.1128/mbio.03883-25 (PMC12977542; doi:10.1128/mbio.03883-25)
Supplement: Supplemental figures — Fig. S1-S3. [file mbio.03883-25-s0001.pdf]

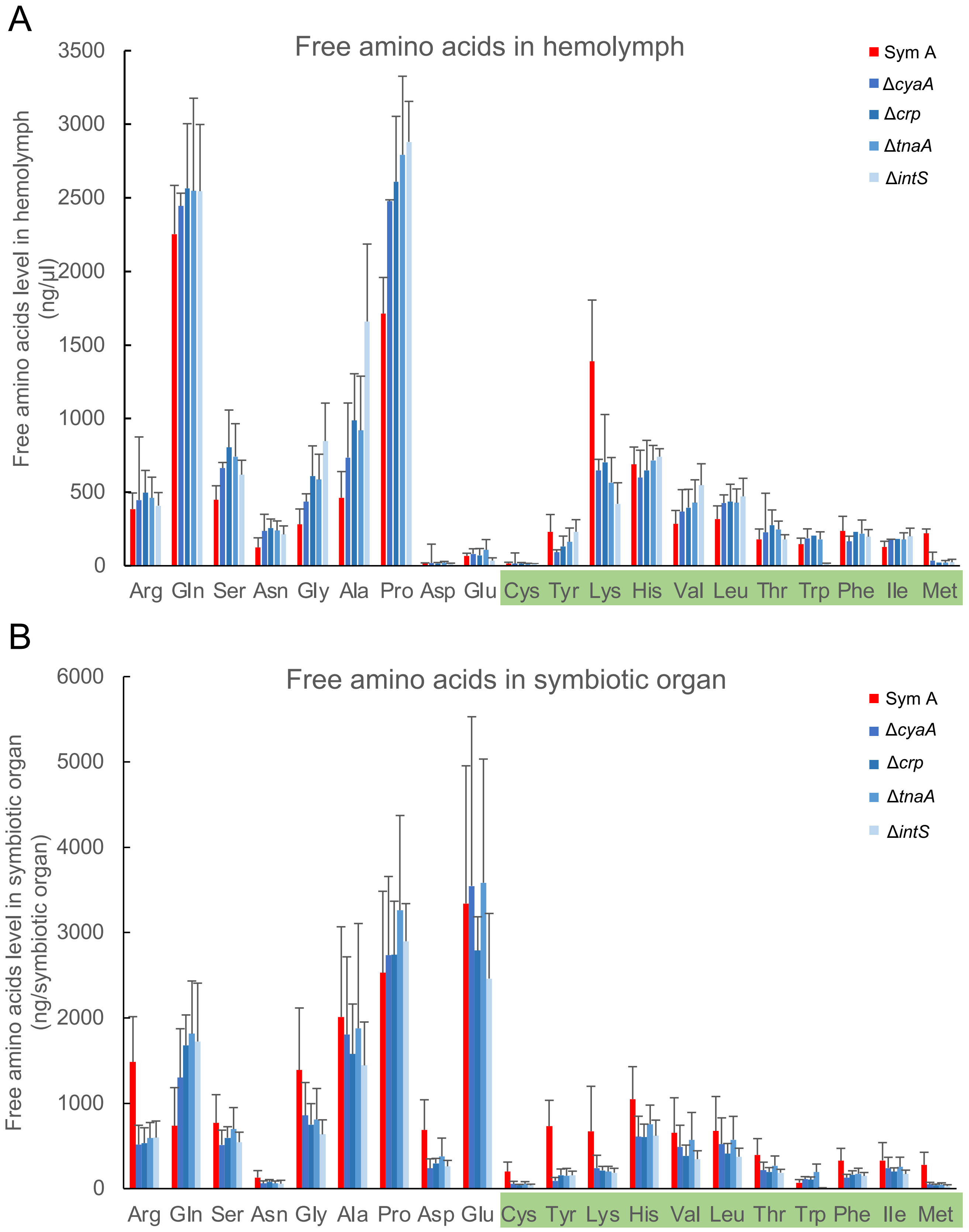


**FIG S1** Levels of free amino acids in hemolymph (A) and symbiotic organ (B) of *P. stali* infected with the natural symbiont *Pantoea* sp. A (SymA), the mutualistic mutant *E. coli* strains (Δ*cyaA*, Δ*crp* and Δ*tnaA*), and the control non-symbiotic *E. coli* strain (Δ*intS*). Means and standard deviations are shown (n = 11-12 each; see Fig. 1). Green shade highlights essential amino acids.

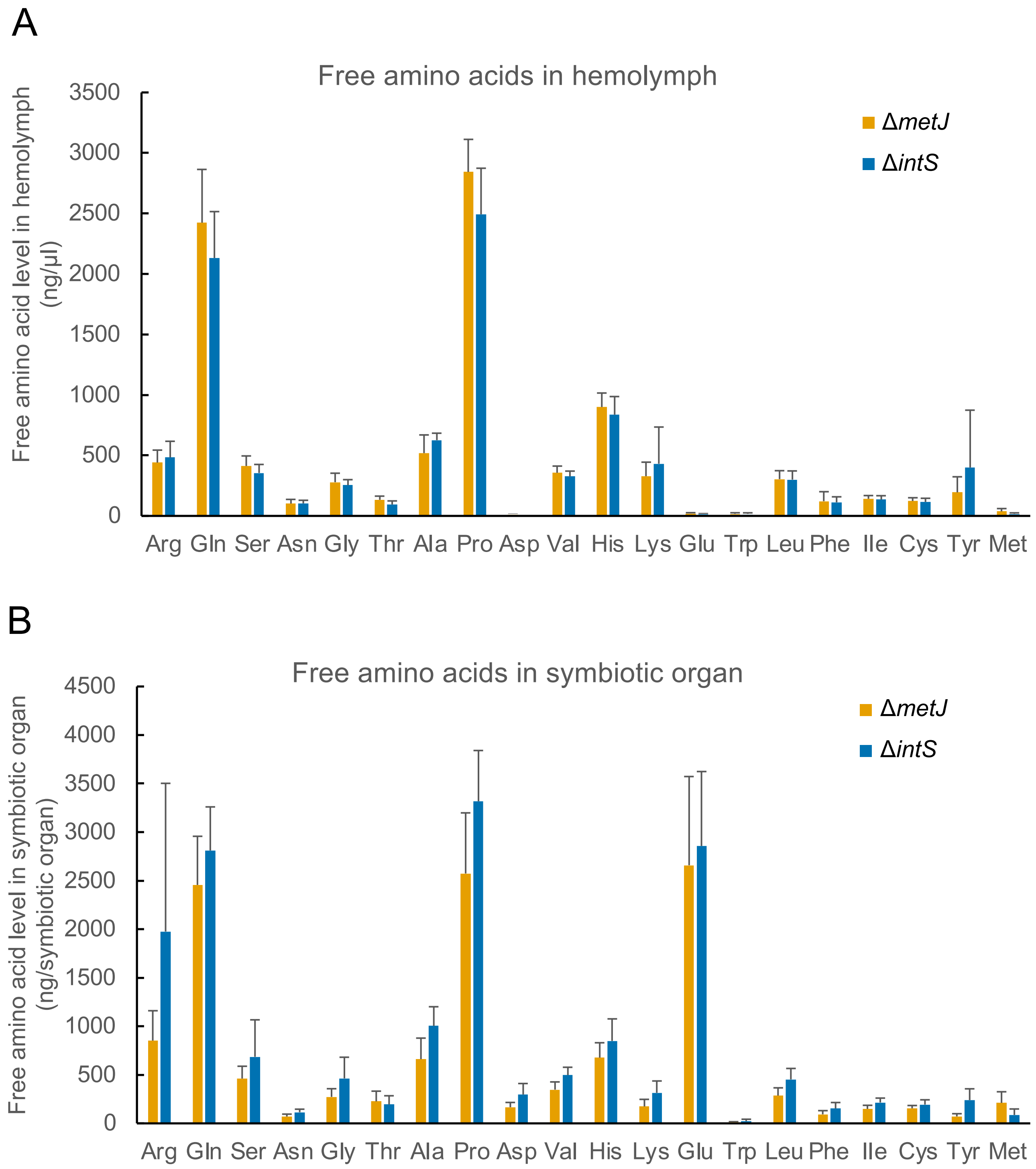


**FIG S2** Free amino acid levels in adult insects of *P. stali* infected with the *E. coli* strains *ΔmetJ* and Δ*intS*. (A) Free amino acids in hemolymph. (B) Free amino acids in symbiotic organ. Means and standard deviations are shown (n = 12 each; see Fig. 3).

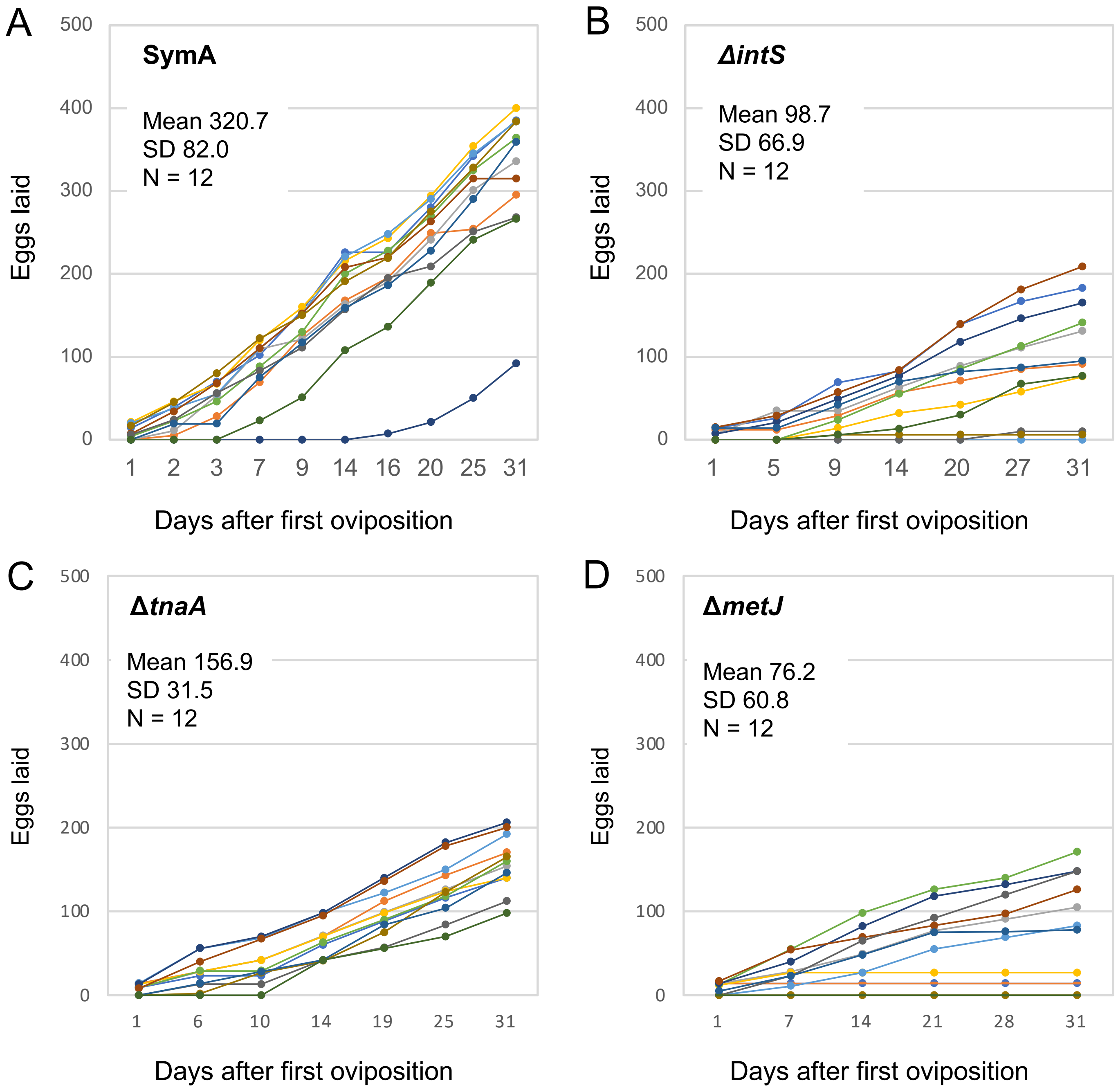


**FIG S3** Cumulative plots of the number of eggs laid by each female during 31 days after emergence. (A) SymA-infected females. (B) Δ*intS*-infected females. (C) Δ*tnaA*-infected females. (D) Δ*metJ*-infected females.
